# A prospective clinical trial on sorafenib treatment of hepatocellular carcinoma before liver transplantation

**DOI:** 10.1186/s12885-019-5760-8

**Published:** 2019-06-11

**Authors:** Malin Sternby Eilard, Mats Andersson, Peter Naredi, Charalampos Geronymakis, Per Lindnér, Christian Cahlin, William Bennet, Magnus Rizell

**Affiliations:** 1 Transplantation Center, Sahlgrenska University Hospital, Gothenburg, 413 45 Gothenburg, Sweden; 2 Department of Surgery, Institute of Clinical Sciences, Sahlgrenska Academy, University of Gothenburg, Gothenburg, Sweden; 3 Department of Radiology, Sahlgrenska University Hospital and Sahlgrenska Academy, University of Gothenburg, Gothenburg, Sweden; 4 Department of Surgery, Sahlgrenska University Hospital, Gothenburg, Sweden

**Keywords:** Liver cancer, Hepatocellular carcinoma, Liver transplantation, Neoadjuvant, Sorafenib, Feasibility, Perfusion CT

## Abstract

**Background:**

Patients with hepatocellular carcinoma waiting for liver transplantation are commonly treated with locoregional treatments, such as TACE and ablation, to prevent tumor progression and dropout and to improve long-term outcome after transplantation. We wanted to prospectively assess feasibility of systemic antitumor treatment with sorafenib as neoadjuvant treatment for hepatocellular carcinoma while waiting for liver transplantation, evaluating tolerability, toxicity and posttransplant morbidity. We also wanted to evaluate perfusion CT parameters to assess tumor properties and response early after start of sorafenib treatment in patients with early hepatocellular carcinoma.

**Methods:**

Twelve patients assigned for liver transplantation due to hepatocellular carcinoma, within the UCSF and who fulfilled other criteria, were included January 2012–August 2014. After baseline evaluation, sorafenib treatment was started. Treatment was evaluated by perfusion CT at 1, 4 and 12 weeks and thereafter every 8 weeks. Toxicity and quality of life was assessed at 1 and 4 weeks and every 4 weeks thereafter during treatment. Treatment was stopped when patients were prioritized on the transplantation waiting list or when intolerable side effects or tumor progress warranted other treatments. Posttransplant morbidity after 90 days was registered according to Clavien-Dindo.

**Results:**

Baseline perfusion CT parameters in the tumors predicted the outcome according to RECIST/mRECIST at three months, but no change in CTp parameters was detected as a result of sorafenib. Sorafenib as neoadjuvant treatment was associated with intolerability and dose reductions. Therefore the prerequisites for evaluation of the sorafenib effect on both CT parameters and tumor response were impaired.

**Conclusions:**

This study failed to show changes in CTp parameters during sorafenib treatment. Despite the curative treatment intention, tolerability of neoadjuvant sorafenib treatment before liver transplantation was inadequate in this study.

**Trial registration:**

EudraCT number: 2010–024306-36 (date 2011-04-07).

**Electronic supplementary material:**

The online version of this article (10.1186/s12885-019-5760-8) contains supplementary material, which is available to authorized users.

## Background

Liver transplantation is the only radical treatment for hepatocellular carcinoma (HCC) that also treats the underlying liver disease. However, even in patients with limited tumor burden who are within the Milan criteria, tumor recurrences after transplantation hamper long-term survival. Long waiting times for liver transplantation may lead to tumor progress beyond accepted criteria, although it has been suggested that a certain time period of observation might be beneficial to exclude tumors with unfavorable biology [1]. Neoadjuvant locoregional treatments such as ablation and transarterial chemoembolization (TACE) have become standard of care in patients waiting for liver transplantation to decrease the risk for tumor progress while on the waiting list, but also to prevent tumor recurrences after transplantation [2]. However, the level of evidence is limited [3] and whether this strategy is merely another selection tool, or actually has a therapeutic effect is not clear [4]. Complete response after locoregional therapy has been demonstrated to decrease the rate of posttransplant tumor recurrences [5–7], while incomplete response and the need for many TACE treatments have been associated with tumor recurrences [8, 9]. Sorafenib, an oral multi-kinase inhibitor, was the first systemic treatment shown to improve survival in the palliative setting of HCC [10, 11]. The drug has antiangiogenic effects as well as direct antitumor effects, but tumor shrinkage measured as response according to RECIST criteria is rarely seen [12]. CT perfusion (CTp) parameters have been suggested as potential biomarkers of response to antiangiogenic drugs because of the ability to quantify tumor perfusion parameters non-invasively [13–16]. Sorafenib-induced tumor perfusion changes probably occur very early after the start of sorafenib treatment [17–19] and could perhaps provide an early prognosis of tumor response.

We hypothesized that sorafenib could improve preoperative treatment by adding a systemic tumor control before liver transplantation and simultaneously prevent local progress.

A theoretical model, based on an assumed antitumor effect also in the early stages of tumor development, suggested that sorafenib would be cost-effective in T2 HCC patients awaiting liver transplantation [20], although there have been concerns about the use of antiangiogenic medication in the perioperative setting [21].

The aim of this study was to evaluate whether the therapeutic effects of sorafenib can be evaluated with CT perfusion parameters and whether sorafenib treatment is feasible in patients with HCC awaiting liver transplantation.

## Methods

**Fig. 1:**
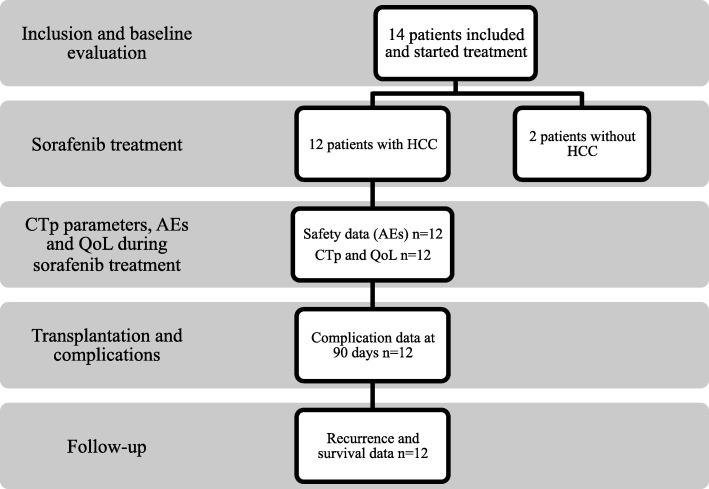
Chart of analyses

This was an exploratory, investigator-driven, non-randomized pilot study, performed at Sahlgrenska University Hospital, Sweden, with inclusion between January 2012 and August 2014. The study was performed according to GCP standards, with study variables documented in case record forms (CRF) and with external monitoring of the study (Gothia Forum, Gothenburg, Sweden). Study termination criteria were set as the need for retransplantation or postoperative death in two patients. Study flow chart is demonstrated in Fig. 1. The study protocol was approved by the Ethical Review Board in Gothenburg, ref. 053–11, and complementary ethical approval regarding survival was approved in 2017 (Dnr T742–17/053–11). EudraCT number: 2010–024306-36 (date 2011-04-07).

### Power calculation and sample size

This study was designed to detect differences in CTp parameters, which was the primary end-point. We calculated that changes of 30% with a subject standard deviation of 30 and 90% power with a two-sided Wilcoxon Signed Rank test, at significance 0.05, would be detected with a study population of 14 patients. Inclusion of another 7 patients after an interim analysis was originally planned, but cancelled because of the low rate of tolerability and the subsequent difficulties interpreting CTp results.

### Study population

Patients were eligible if assigned for liver transplantation due to HCC by the multidisciplinary conference with an expected waiting time exceeding three months. Inclusion criteria included HCC diagnosis based on histology or in accordance with the non-invasive European Association for the Study of the Liver (EASL) criteria [22], tumor burden within the UCSF criteria [23] and informed consent. Additional inclusion criteria were preserved liver function, measured as Child-Pugh < 8 and acceptable laboratory values defined as Hb > 9.0 g/%, WBC > 3000 cells/mm^3^, ANC > 1500 cells/mm^3^, platelets > 50,000 cells/mm^3^, liver function tests with Bilirubin < 3 mg/dl, PT-INR/PTT < 1.5 x ULN and renal function with serum creatinine < 1.5 x ULN, or a measured GFR > 60 ml/m^2^. Furthermore, ECOG performance status of no more than 1 was required. Exclusion criteria were macroscopic vascular invasion, extrahepatic tumor spread, any prior HCC treatment, ongoing infection, age less than 18, significant cardiovascular disease, severe pulmonary disease, uncontrolled hypertension, thrombotic or embolic events including transient ischemic attacks within the past six months or previous malignancy, organ transplantation, ongoing pregnancy, HIV, immunosuppressive treatment, or mental conditions rendering the patient incapable of understanding the consequences of the study.

During the inclusion period a total 46 HCC-patients were transplanted. In 15 patients inclusion criteria were not fulfilled due to Child score more than 7 and another three were outside the UCSF. Some patients were not judged eligible because of an estimated waiting time less than three months, while a few patients preferred standard treatment and declined study participation.

### Endpoints

Primary endpoint: Changes in perfusion of tumors as measured by CTp. Secondary endpoints: Tumor response according mRECIST, percentage of patients reaching liver transplantation, percentage of patients with radiographic tumor progression, impact of sorafenib on quality of life, impact of sorafenib on liver function and toxicity during waiting list time according to CTC v4.0, postoperative morbidity according to Clavien-Dindo and mortality.

### Treatment

Sorafenib was initiated at full dose (400 mg bid). Clinical and laboratory monitoring and quality-of-life assessments were carried out at one and four weeks and thereafter every four weeks. Dose modifications, temporary treatment pauses and symptomatic treatments were prescribed depending on side effects. To ensure at least one week off medication before transplantation study treatment was stopped when subjects were given high priority on the waiting list, which was discussed on a weekly basis depending on the current waiting list situation.

### Adverse events

Common toxicity criteria (CTC) v4.0 was used for adverse events registration during sorafenib treatment. Surgical morbidity and mortality 90 days after liver transplantation was evaluated and classified according to the Clavien-Dindo scale [24].

### Quality of life (QoL)

QoL was evaluated by the questionnaires EORTC QLQ C30 and HCC18. The questionnaires include single and multiple items that were grouped into global health, functional domains and symptom scales. Domains, scales and single items were converted to scores ranging from 0 to 100 according to the EORTC scoring manual [25]. For QLQ C30 functional or global scores, higher scores represent a healthier level of functioning, whereas for symptom scales higher scores represent more severe symptoms.

### Response evaluation

CT was performed at baseline, and repeated at 1, 4 and 12 weeks and thereafter every 8 weeks. CT examinations were performed according to a protocol for CT perfusion [26], (see Additional file 1: CT protocol). CTp enables analysis of the temporal changes of tissue and vessel attenuation after intravenous injection of iodinated contrast medium. The perfusion parameters used were Blood Flow (BF), the rate of blood passing through the vascular bed in a given tissue location (mL/100 g/min). The Mean Transit Time (MTT) reflects the average time it takes for the blood to pass from the arterial entry to the venous exit (seconds). Blood Volume (BV) is computed as the product of BF and MTT (mL/100 g). The Hepatic Arterial Fraction (HAF) represents the proportion of liver blood input supplied by the hepatic artery (a value between 0 and 1). The arterial perfusion of the tumors (AF) is the product of BF and HAF. Finally, we measured the permeability surface area product (PS), which is a surrogate measure of vascular leakiness (mL/100 g/min), representing the extraction of solutes from the blood plasma to the interstitial space.

Radiological tumor response during treatment with sorafenib was assessed according to mRECIST [27]. Up to two lesions in the liver, at least 1 cm in size, were selected and the largest diameters (LD) of intratumoral arterial enhancement were measured and recorded. If sufficient arterial enhancement was not present, measurements of the longest overall tumor diameter according to conventional RECIST were used [28].

### Survival and tumor recurrences

Survival and recurrence data was updated August 30, 2017. Date of death was checked in the population registry.

### Statistics

For perfusion data the mean and standard deviation of BV, BF, MTT, HAF, AF and PS measurements were determined. The Wilcoxon Rank Sum test was used for comparison of CTp parameters at baseline and one week and between the index lesion and the liver parenchyma. Comparison between CTp parameters at baseline and the percentage change after one week and the RECIST/mRECIST at 12 weeks was performed with the Mann–Whitney U test. Spearman correlation analysis was performed for each of the perfusion parameters and the rate of tumor growth at 12 weeks. Intra-observer agreement was assessed using the one-way intraclass correlation coefficient (ICC). Values between 0.60 and 0.74 were interpreted as good and values between 0.75 and 1.0 were interpreted as excellent [29]. Differences with *p* values of < 0.05 were considered statistically significant. Analyses of CTp parameters were performed using the software WinSTAT plug-in for Excel (Microsoft Office 2010) and Medcalc (Medcalc Software, Ostend, Belgium).

Descriptive statistics were calculated using Microsoft Excel for Mac 2011, version 14.7.2. QoL-analysis was performed despite single values missing in domains with at least three questions. The Wilcoxon Rank Sum test was used for QoL comparisons with SPSS v24.

## Results

Patient and tumor data are shown in Table 1. Twelve HCC patients were included and analyzed (nine men and three women). Median age at inclusion was 55 years (range 32–68). All HCC-patients had a tumor burden within the UCSF criteria and 11 were also within the Milan criteria according to pretransplant radiology. In three patients the etiology was a combination of hepatitis C and alcohol. According to explant pathology, eleven patients had cirrhosis while one patient with hepatitis B had fibrosis.

**Table 1:** Demographics

Parameter	Definition	Average N (%)	[range]
Gender	Female/Male	3/9 (25/75)	
Age at inclusion (years)	Median	55	[32–68]
BMI (kg/m^2^)	Median	29	[25–35]
Etiology^a^	Hepatitis C	8 (67)	
	Hepatitis B	2 (17)	
	Alcohol	5 (42)	
Comorbidity^a^	Hypertension	4 (33)	
	Diabetes	2 (17)	
	Pulmonary disease	1 (8)	
	No comorbidity	6 (50)	
ECOG at inclusion	0	7 (58)	
	1	5 (42)	
Child–Pugh score at inclusion	5	5 (42)	
	6	6 (50)	
	7	1 (8)	
MELD^b^ score at inclusion		9	[6–13]
AFP level at inclusion (mg/L)	Baseline median	22.5	[2.3–2790]
	< 100	9 (75)	
	≥ 100	3 (25)	
Criteria at inclusion	Within Milan	11 (92)	
	Within USCF	12 (100)	
Tumor number at inclusion^c^	1	7 (58)	
	2	1 (8)	
	3	4 (33)	
Longest tumor diameter at baseline^d^ (cm)	mRECIST median	2.5	[1.5–4.4]
Total diameter at baseline^d^ (cm)	mRECIST median	2.6	[1.8–6.3]
Explant histology			
Longest diameter (cm)	Median	4.3	[0.8–6.5]
Total diameter (cm)	Median	5.0	[1.3–12.2]
Tumor number	1/2/3/> 3	5/3/1/3	
Vascular invasion	Macro/Micro/None/undef	1/7/3/1	
Differentiation grade (Edmonson)	2/3/4/undef	2/8/1/1	
Mixed type HCC	Cholangiocellular diff	1 (8)	

^a^patients could have more than one factor, ^b^MELD score including Na, ^c^refers to screening radiology, ^d^one patient could not be evaluated according to mRECIST (*n* = 11)

Two additional patients were included, but excluded from analyses since HCC was not confirmed. The first received only one dose of sorafenib and explant pathology showed neuroendocrine cancer. In the second patient, the malignant diagnosis was questioned upon repeated CT scans during sorafenib treatment and was not transplanted.

### Sorafenib medication

Patients started sorafenib treatment after a median of one day from inclusion (range 0–7). Patients were on sorafenib treatment for a median of 155 days (range 4–365) including temporary treatment pauses, which corresponds to a median of 30% and a mean of 46% (range 2–99%) of the waiting list time; Table 2. Median time with active treatment was 103 days (range 4–326). Mean and median dose was 474 and 400 mg per day respectively. At 12 weeks, five patients had stopped the sorafenib treatment.

**Table 2:** Treatment and responses

ID	Sorafenib treatment time (weeks)	Mean daily dose (mg)	Response during sorafenib	Time to tumor progress^a^ while waiting for Ltx (weeks)
1	52.1	382	PD	28
2	5.1	433	SD	20
3	23.3	145	PD	12
4	21.0	524	SD	–
5	23.1	431	PD	20
6	6.0	210	SD	12
7	23.3	798	PD	12
8	27.1	412	PD	20
9	11.4	230	SD	–
10	0.6	700	SD	–
11	3.3	522	SD	–
12	24.3	561	SD	–

^a^according to mRECIST

Treatment duration, mean daily dose, treatment response during sorafenib treatment and time to tumor progression while waiting for transplantation for each individual

**Fig. 2:**
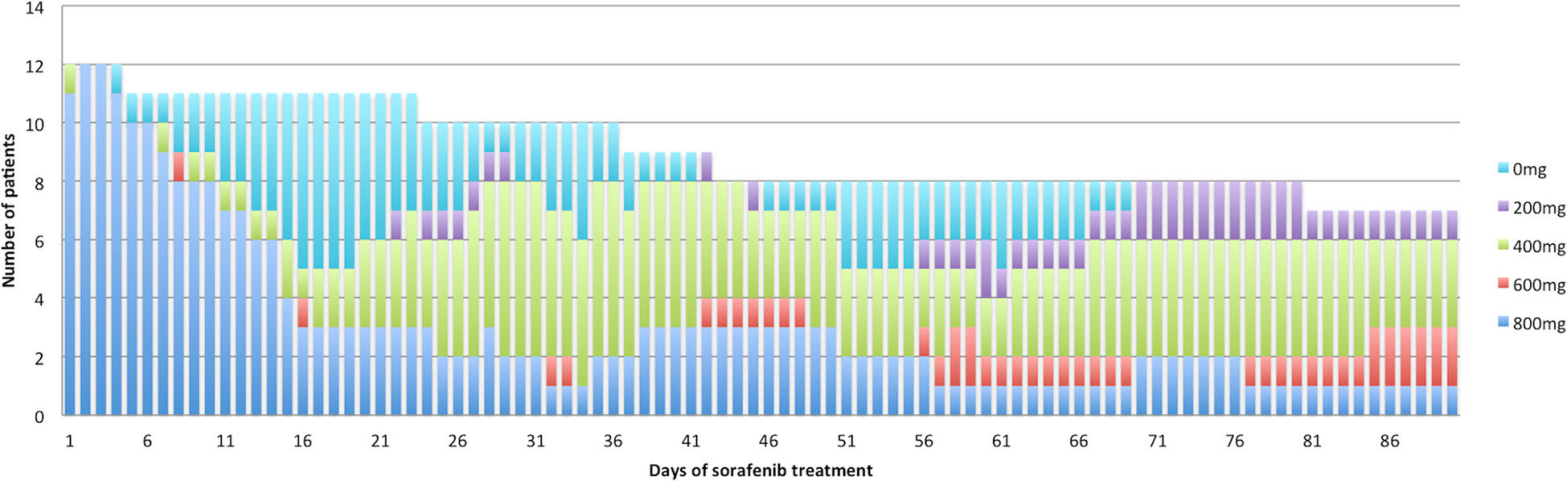
Daily treatment dose. Number of patients on each dose of sorafenib during the first 90 days of treatment. 0 mg corresponds to patients with a pause in treatment at the time, but who had not stopped treatment permanently

Ten patients needed dose modifications and 9 required up to four treatment pauses; Fig. 2. Six patients terminated treatment because of side effects/adverse events, including hand keratosis, liver enzyme deviation, leucopenia, diarrhea, abdominal pain, deteriorated vision, fever, tremor, fatigue. Three patients discontinued sorafenib because of HCC progression, of which one had also reached priority on the waiting list. Another three patients stopped sorafenib treatment only because they had reached priority on the waiting list. Patients were off sorafenib treatment for a median of 86 days (range 4–462) before transplantation. Rescue treatment with TACE was given in seven patients after a median of 29 days from sorafenib stop (range 14–103). One patient received two TACE treatments.

### Tumor perfusion

The perfusion parameters Blood Flow (BF) and Arterial Blood Flow (AF) of the HCC lesions were significantly higher than the respective parameters of liver parenchyma (*p* < 0.05 and *p* < 0.01). No significant differences were found regarding Blood Volume (BV), Mean Transit Time (MTT) and Permeability Surface (PS); Table 3.

**Table 3:** CT perfusion measurements

CTp measurements	Index tumor at baseline (mean ± SD)	Liver at baseline (mean ± SD)	*P* value	Mean % change index tumor 1w	*P* value	Mean % change liver 1w	*P* value
BV (ml/100 g)	23.1 ± 8.0	21.3 ± 12.4	0.60	−10.0	0.16	−19.1	0.06
BF (ml/100 g/min)	184.7 ± 141.1	102.6 ± 46.4	< 0.05	−10.9	0.16	−14.9	0.05
MTT (seconds)	13.8 ± 6.9	20.4 ± 9.7	0.08	11.5	0.94	−7.5	0.53
HAF (in %)	46.1 ± 21.1	31.6 ± 22.8	0.11	−13.4	0.27	47.4	0.30
AF (ml/100 g/min)	84.2 ± 77.0	30.1 ± 21.1	< 0.01	−16.9	0.10	19.1	0.69
PS_Index_ (ml/100 g/min)	26.0 ± 22.2	37.0 ± 26.0	0.30	59.8	0.31	52.1	0.18

CT perfusion measurements in the index tumor and in background liver before treatment start and after one week (*n* = 12)

After one week of sorafenib treatment the BV, BF and Hepatic Arterial Fraction (HAF) showed lower mean values and MTT and PS showed higher mean values than at baseline in tumors as well as in the liver parenchyma, but the differences were not statistically significant; Table 3. At the 4- and 12-week time points the perfusion parameters tended to regress to the baseline values.

**Fig. 3:**
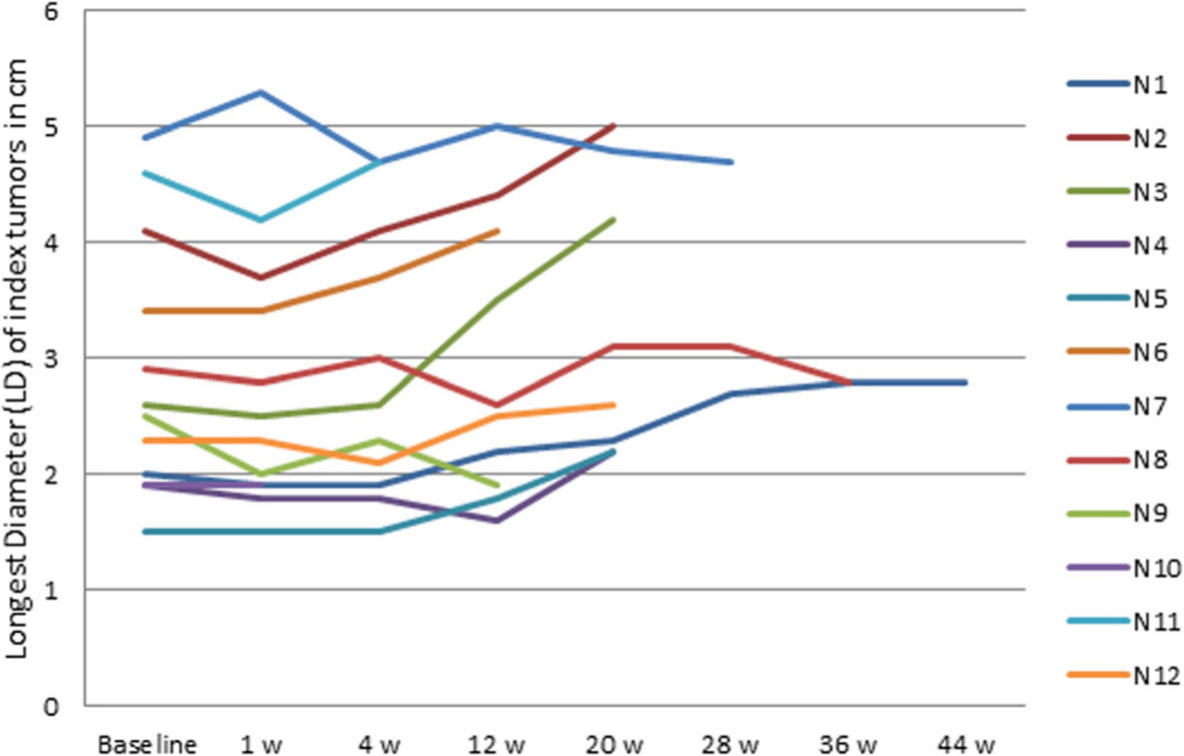
Tumor diameter. Change in longest diameter (LD) of index tumors at follow up CTp examinations (measured in cm according to RECIST) (*n* = 12)

At 12 weeks after start of treatment, response evaluation according to RECIST and mRECIST showed stable disease (SD) in seven patients. In three patients the evaluation revealed progressive disease (PD) according to RECIST/mRECIST. Patients with PD had significantly lower mean BF and AF at baseline than patients with SD (80.5 ± 13.3 vs 241.3 ± 162.4 ml/100 g/min for BF and 27.1 ± 18.9 vs 105.0 ± 92.0 for AF, p < 0.05). No other CTp parameter correlated with response according to RECIST/mRECIST. The mean tumor LD (Longest Diameter) was 2.8 ± 1.1 cm at baseline and 3.0 ± 1.1 cm at 12 weeks; Fig. 3. No correlation between baseline CTp parameters or change of these after one week and the percentage growth in LD after 12 weeks was found. Median time to progression according to RECIST and mRECIST was 20 weeks in both cases.

### Adverse events and liver toxicity

Median number of adverse events per patient was 10 (range 4–13); Table 4. There was no serious adverse event. A total of eight grade 3 adverse events were registered in three patients. The Child–Pugh score varied during the treatment period, but there was no more than a two-point increase in a single patient and never a score higher than 8. No significant deterioration in lab parameters was seen during treatment (Additional file 2: Table Lab parameters).

**Table 4:** Adverse events

Type of adverse event	Total N AEs/ subjects	Reason stop	Reason modi-fication	AEs CTC gr 3	AEs CTC gr 2	AEs CTC gr 1	Median days to AE (range)
Leucopenia	**2/2**	**1**	**–**	**0**	**1**	**1**	**51 (13–88)**
GI disorders	**17/7**	**2** ^**b**^	**4**	**2**	**9**	**6**	
*Diarrhea*	12/7	2^b^	4	2	6	4	61 (0–305)
*Nausea*	3/3				1	2	4 (0–7)
*Ulcer*	2/2				2		65 (7–123)
General disorders	**35/11**	**3** ^**b,c**^	**10**	**4**	**13**	**18**	
*Fatigue*	9/9	1	3	1	3	5	7 (0–54)
*Loss of appetite*	7/6		1	1	2	4	7 (3–139)
*Weight problem*	2/2					2	65 (15–112)
*Pain*	11/8	2^a,b,c^	4	2	2	7	11 (0–139)
*Hypertension*	6/6		1		6		18 (7–193)
Hepatobiliary disorders	**5/4**	**1**	**3**	**0**	**2**	**2**	**7 (6–85)**
Dermatologic disorders	**31/10**	**1**	**7**	**1**	**12**	**18**	**15 (4–145)**
Other	**33/11**	**3** ^**c**^	**2**	**1**	**12**	**20**	
*Hoarseness*	2/2					2	17 (13–21)
*Eyes and Vision*	5/4	1^c^	1	1	2	2	9 (3–61)
*Headache*	2/2				2		–
*Infection/fever*	6/6	1^c^			4	2	13 (3–167)
*Other related*	8/5	1^c^	1		3	5	–
*Other not related*	10/7				1	9	
Serious adverse event	0/0	0	0	0	0	0	
TOTAL events/patients:	123/ 12			8/ 3	49/12	65/11	

^a^abdominal pain, ^b^one patient had dual reasons for stopping treatment, ^c^several adverse events led to treatment stop in one patient

Adverse events (AEs) according to CTC v4.0 during sorafenib treatment. ‘Reason stop’ refers to the number of patients who stopped sorafenib treatment permanently because of an adverse event, while reason ‘Reason modification’ corresponds to the number of times an adverse event led to a treatment pause or dose modification. Some adverse events occurred several times in the same patients, which is why the total number of AEs is higher. The subgroups of Gastrointestinal disorders, General disorders and Other are in italics

Bold figures are the sums of the subgroups (in italics)

### Quality of life

**Fig. 4:**
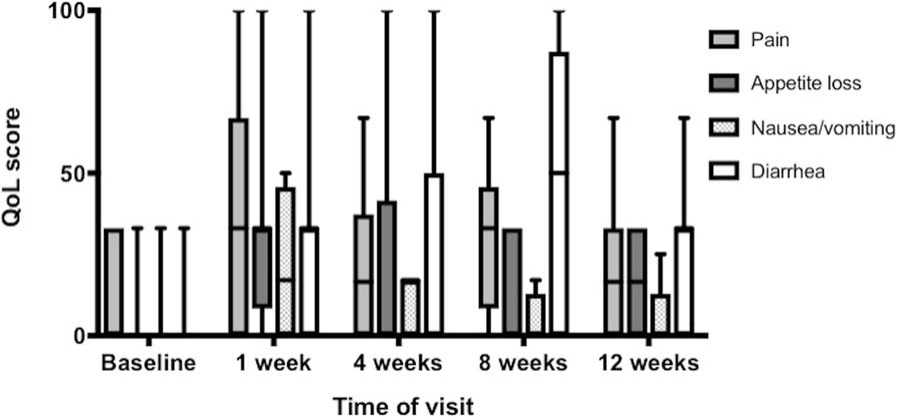
QoL C30 symptom scores. Boxplots of QLQ C30 scores in patients on sorafenib treatment during the first 12 weeks according to four domains of the EORTC quality of life questionnaire C30. The box represents the interquartile range, the band inside the box is the median and the whiskers represent range

Only QoL data from patients who were still on treatment were included in the analysis at each time point. Quality-of-life scores varied considerably between patients, but a pattern with worst mean values at one week after treatment start was observed in several domains. The change from baseline was significant for C30 Nausea and Vomiting (*p* = 0.043); Appetite Loss (*p* = 0.008) and Pain score (*p* = 0.045); Fig. 4. A similar non-significant pattern was seen for C30 Global health, the Physical, Social, Role and Cognitive functioning scales and the HCC18 domains Fatigue, Fever and Pain. Though the changes were not significant, the HCC18 Nutrition scale indicated the highest symptom burden at 4w, while C30 Diarrhea peaked at 8w.

### Transplantation and complication rates

All 12 HCC patients underwent liver transplantation. Median time from HCC diagnosis and from inclusion to liver transplantation was 294 days (range 194–583) and 231 days (range 81–515) respectively. Median time from stopping sorafenib treatment to liver transplantation was 86 days (range 4–462).

Within 90 days from liver transplantation, 11 patients (*n* = 12) had a complication grade 1 or worse according to Clavien-Dindo; Table 5 [24]. In two patients, the worst observed complication was grade 1, while grade 2 was the worst in seven patients. In two patients the most serious complication was grade 3b as they developed pseudoaneurysms of the hepatic artery, which were treated surgically. Both had been treated for rejection and endoscopically for bile leakage. A third grade 3b complication, a cardiac arrhythmia, was treated by electroconversion. No primary non-function was seen. Median hospital stay was 12 days.

**Table 5:** Posttransplant complications

Complication	Grade 1	Grade 2	Grade 3a	Grade 3b
Rejection		2		
Bile duct complication			2	
Vascular complication				2
Wound complication	3			
Bleeding			1	
Infection		2		
Heart				1
Gastrointestinal	1	1		
Kidney	2			
Ascites		1		
Other		6		

Number of patients with each postoperative complication within 90 days after liver transplantation. Grades are according to Clavien-Dindo classification of surgical complications. There were no grade 4 or 5 complications

### Survival and recurrence

**Fig. 5:**
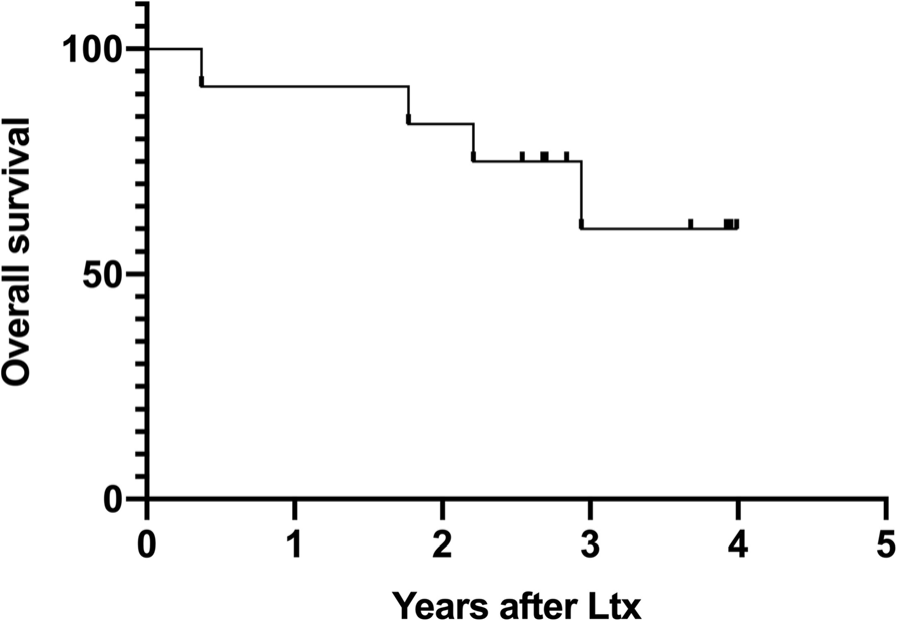
Overall Survival after liver transplantation. Estimated overall survival after liver transplantation according to Kaplan-Meier (*n* = 12)

Mean follow-up time among living patients was 1200 days. Two years’ survival was 83% and estimated three- and five-year survival rates according to Kaplan-Meyer were 75 and 56% respectively, Fig. 5. The mean time to recurrence among the five patients with recurrence was 450 days.

## Discussion

To our knowledge, this is the first prospective study aiming to evaluate the feasibility of neoadjuvant sorafenib as a single therapy while waiting for liver transplantation. However, the study failed to show a correlation between changes in CTp parameters during sorafenib treatment and mRECIST response.

We found that baseline CT tumor perfusion parameters were associated to tumor response at three months. Using CTp, we found that the Blood Flow and Arterial Blood Flow were significantly higher in the HCC tumors compared to the surrounding liver parenchyma. Baseline perfusion in the tumors predicted the outcome according to RECIST/mRECIST at three months. Patients with progressive disease (PD) had significantly lower baseline BF and AF than patients with stable disease (SD) (there were no cases of partial or complete response). It has been reported that patients with low tumor angiogenesis may have a worse outcome than those with higher tumor angiogenesis [17, 30, 31]. Patients with higher arterial tumor flow (pre-AF_Tumor_ higher than 71,7 ml/min/100 ml) had better overall survival than patients with lower pre-AF_Tumor_ and a decrease in AF after one week tended to be associated with survival [17]. In contrast, we did not find any significant decrease in tumor perfusion after one week of sorafenib treatment. Neither did we find any association between the change in perfusion parameters at one week and the response according to RECIST/mRECIST, rate of tumor growth or of tumor recurrence in the transplanted liver.

For HCC patients treated with sorafenib the standard of clinical practice is response evaluation according to the mRECIST criteria [32]. Due the frequent dose modifications and treatment interruptions the assessment of responses from sorafenib treatment in this cohort was severely impaired. The fact that only seven patients were still on treatment at 12 weeks makes it difficult to evaluate the sorafenib treatment effect on the CTp parameters during the continuation of the treatment up to and beyond the 12-week time point. In addition, seven patients received complementary TACE treatments to prevent tumor progression.

We found that the time to progression according to mRECIST was 20 weeks, which is within the same range as the reported 5.5 months median time to progression in HCC patients treated with sorafenib [10]. In previous studies in advanced tumor stages, the survival benefit described with sorafenib was seen despite a lack of radiologic response. The results of an adjuvant phase III trial, published in 2015 [33], where sorafenib after liver resection and ablation was studied in 1114 patients, were not known at the time when our study was designed. No benefit with sorafenib in the adjuvant setting was demonstrated in this study.

Considering feasibility of sorafenib as a pretransplant treatment, the results were disappointing regarding tolerability (toxicity and impact on quality of life during sorafenib treatment on the waiting-list). This study was not designed to evaluate the efficacy of sorafenib preventing tumor progression during treatment and posttransplant recurrences, and with the frequent dose modifications, such assessments were even more precarious and no conclusions regarding efficacy can be drawn.

We expected this selected patient cohort with good performance status, early tumor stage and low comorbidity to tolerate the side effects of sorafenib better than a palliative cohort and therefore started with full-dose treatment. The difficulty in keeping these patients on sorafenib treatment was unexpected, since we had a robust previous experience with sorafenib treatment in palliative patients. Half of the patients terminated sorafenib medication because of side effects, such as diarrhea and different kinds of dermatologic problems, concordant with previously reported side effects [10, 34, 35]. In a randomized study where TACE-treated patients received the addition of either sorafenib or placebo, there were more objective responses and fewer dose modifications, but there were also more severe adverse events [34]. In our study the mean daily dose was 474 mg during the treatment period and no patient in this study could continued full-dose treatment without interruption or dose modifications, similar to findings in another neoadjuvant sorafenib study [35].

The negative impact of sorafenib on quality of life was reflected by a significant deterioration at one week. Despite early tumor stages, pain was observed to be an issue both among AE reports (11 related pain events among eight patients) and in the C30 Pain score.

When this study was planned there were only a few case series/reports on sorafenib treatment before liver transplantation [36–38]. High complication rates have been reported in patients receiving sorafenib before transplantation [35, 39], but no firm conclusions can be drawn due to the small sample sizes, and other reports showed no increased complication rate [40]. In our study, the rate of postoperative complications 90 days after transplantation was high, but in parity with rates after transplantation for tumors of the liver and bile ducts at 30 days in the Swedish national registry (SweLiv) [41]. The most serious complications in this study were two cases of pseudoaneurysm. It is unlikely that sorafenib had any impact on the complications in these two patients, since they had been off sorafenib treatment for 149 and 169 days respectively before liver transplantation.

Due to the limited sample size, we did not intend to analyze the rate of tumor recurrence in this study. However, the rationale for using sorafenib during waiting-list time resides in its potential to prevent recurrence. Despite that 11 out of 12 patients were within Milan criteria at baseline, 5 had tumor recurrence at follow-up, which is discouraging. However, with the small number of patients, the low tolerability with frequent dose reductions and long periods without treatment, along with a large proportion of rescue TACE treatments, no conclusions regarding the efficacy of sorafenib can be drawn in this study. The high rate of treatment modifications is an important limitation of this study, which not only restrict the interpretation of results concerning CTp but also secondary endpoints. The variation in time from stopping sorafenib treatment to liver transplantation impairs the interpretation of sorafenib treatment as a cause of posttransplantation complications.

The CTp in this study also has several limitations. A major limitation was the lack of motion correction. It has been shown that within an individual patient, only a decrease of more than 35% for BF can be considered beyond the variability related to the breath-hold CTp analysis process in HCC used in our study [42]. However, our acquisition method complied with the current international guidelines [26] and the ICC between readers of the perfusion parameters in the tumors and in the liver parenchyma was good (ICC 0.60 and 0.78, respectively).

## Conclusion

We found that the CTp parameters baseline Blood Flow (BF) and Arterial Blood Flow (AF) in the tumors predicted the outcome according to RECIST/mRECIST at three months. The study was negative for detecting changes in CTp parameters during sorafenib treatment. CTp is a technique under development that may become useful for assessing treatment response, but CTp of the liver can only be recommended if motion correction software is available. In this study, sorafenib as neoadjuvant treatment was associated with intolerability and dose reductions.

## Additional files

Additional file 1: CT protocol. Detailed description of the CT perfusion procedure. (DOCX 110 kb)

Additional file 2: Laboratory parameters. Table of the lab parameters during the first 12 weeks of sorafenib treatment. (DOCX 72 kb)

## Data Availability

The datasets used and/or analyzed during the current study are available from the corresponding author on reasonable request.

## Abbreviations

AEs: Adverse events
AF: Arterial perfusion of tumors
AFP: Alfa fetoprotein
ANC: Absolute neutrophil count
BF: Blood flow
BMI: Body mass index
BV: Blood volume
CRF: Case report form
CT: Computer tomography
CTC: Common toxicity criteria
CTp: Computer tomography perfusion
EASL: European association for study of the liver
ECOG: Eastern Cooperative Oncology group
EORTC: European organisation for research and treatment of cancer
GCP: Good clinical practice
GFR: Glomerular filtration rate
GI: Gastrointestinal
HAF: Hepatic arterial fraction
HCC: Hepatocellular carcinoma
HIV: Human immunodeficiency virus
ICC: Intraclass correlation coefficient
LD: Largest diameter
mRECIST: Modified Response Evaluation Criteria in Solid Tumors
MTT: Mean Transit Time
PD: Progressive disease
PS: Permeability Surface Area Product
PT-INR: Prothrombine Time – International Normalized Ratio
QLQ: Quality of life questionnaire
QoL: Quality of life
RECIST: Response Evaluation Criteria in Solid Tumors
SD: Stable disease
SweLiv: Swedish Registry for tumors of the liver and bile ducts
TACE: Transarterial chemoembolization
UCSF: University of California San Fransisco
ULN: Upper limit of normal
WBC: White blood count
