# Supplementary material for: A prospective clinical trial on sorafenib treatment of hepatocellular carcinoma before liver transplantation
Source: BMC Cancer. 2019 Jun 11;19:568. doi: 10.1186/s12885-019-5760-8 (PMC6560824; doi:10.1186/s12885-019-5760-8)
Supplement: Supplementary file 1 — CT protocol. Detailed description of the CT perfusion procedure. (DOCX 110 kb) [file 12885_2019_5760_MOESM1_ESM.docx]

**Electronic Supplementary Material**

**CT protocol**

Volumetric helical CT perfusion (CTp) was performed on a 64-slice MDCT scanner (Lightspeed VCT, GE Healthcare, Milwaukee, WI, USA). A non-contrast upper abdominal study was performed initially in order to confirm the location of the known liver tumor. For perfusion imaging, 51–59 ml of contrast medium with iodine concentration of 350 mg I/ml was administered intravenously at a flow rate of 5–6 ml/s, followed by 40 ml of saline solution at the same flow rate. A delay time of 7–10 sec from the beginning of the injection of contrast material to the initiation of scanning was applied. Using the shuttle mode, a volume of 11–12 cm in the z axis covering the lesion was scanned. Examination in the first, intravascular phase was performed in two consecutive breath-holds. The patients were trained in the breath-hold technique prior to the examination, and restraining bands were used to limit involuntary respiratory movements of the abdomen. A breathing pause of 4 seconds was allowed after the first 19–25 seconds-long breath-hold. In total 24–27 scans were obtained in consecutive cranio-caudal and caudo-cranial directions during the first phase. The time per pass was 1.5 seconds, tube voltage 100–120 kV, automatic tube current and the reconstructed slice thickness 5 mm. The duration of the first-pass acquisition was 40 seconds and it was followed by six breath-hold scans, separated from each other by 15 seconds, necessary for the computation of the permeability surface area product (PS) parameter. The CT perfusion study was followed by a diagnostic portal venous phase upper abdominal study. This examination started 90 seconds after initiation of injection of 80 ml 350 mgI/ml contrast material at a rate of 3 ml/s. This diagnostic examination was used for staging purposes and reported according to our clinical practice. Data acquisition parameters and the anatomic location for scanning were kept constant for each patient and for each repeat CTp study.

The resulting image material was transferred to a workstation (Advantage Windows 4.4, GE Medical Systems) with the CT Perfusion 4.0, version 4.3.1 software. The software uses ‘deconvolution’ analysis of a distributed parameter model taking capillary permeability into consideration. This allows simultaneous determination of Blood Flow (BF in mL/100 g/min), Blood Volume (BV in mL per 100 g), Mean Transit Time (MTT in seconds), Hepatic Arterial Fraction (HAF as fractional value between 0.0 and 1.0) and permeability surface area product (PS in mL/100 g/min). The model requires placing a region of interest (ROI) in an artery (aorta) and another in the portal vein to obtain representative arterial and venous ‘time attenuation curves’ (TACs). The largest tumor lesion was selected by a radiologist with more than 20 years in liver imaging. An ROI was manually drawn covering the entire tumor target on the CT slice showing the maximum contrast enhancement. In case of mismatch due to motion the ROI was relocated to fit the area with deviant perfusion corresponding to the tumor. The perfusion parameters (BV, BF, MTT, HAF and PS) within the ROIs were automatically derived. The arterial perfusion of the tumors (AF) was then calculated by multiplying the blood flow (BF) with the arterial flow fraction (HAF).

For measurement of background liver, an elliptical ROI was placed in unaffected parenchyma, avoiding vessels and artefacts. To assess interobserver agreement, a second subspecialist-trained radiologist performed image analysis of the perfusion data in the baseline studies of four of the patients.

The mean Dose-Length Product (DLP) value for the CTp was 1089.2 mGy x cm (range 831–1414), whereas the mean DLP for the staging CT of the liver was 520.3 mGy x cm (range 224–1185). Using the standard conversion coefficient for the abdomen (К = 0.018 mSV/(mGy x cm) the calculated mean effective radiation dose was 19.6 mSv and 9.4 mSv, respectively. The mean DLP for the entire study, displayed at the CT console, was 1865.7 mGy x cm (range 1274–2954), corresponding to a mean effective dose of 33.6 mSv.
