# Supplementary material for: A prospective clinical trial on sorafenib treatment of hepatocellular carcinoma before liver transplantation
Source: BMC Cancer. 2019 Jun 11;19:568. doi: 10.1186/s12885-019-5760-8 (PMC6560824; doi:10.1186/s12885-019-5760-8)
Supplement: Supplementary file 2 — Laboratory parameters. Table of the lab parameters during the first 12 weeks of sorafenib treatment. (DOCX 72 kb) [file 12885_2019_5760_MOESM2_ESM.docx]

Supplementary Table Laboratory Parameters

|  | N | Mean base-line | N | Mean 1  week | N | Mean 4 weeks | N | Mean 8  weeks | N | Mean 12  weeks |
| --- | --- | --- | --- | --- | --- | --- | --- | --- | --- | --- |
| Hb (g/L) | 12 | 137 | 12 | 144 | 11 | 138 | 11 | 134 | 10 | 142 |
| WBC (10^9^/L) | 12 | 4.5 | 12 | 4.8 | 11 | 3.8 | 11 | 4.3 | 10 | 4.8 |
| Neutrophils (10^9^/L) | 12 | 2.6 | 12 | 2.8 | 11 | 2.2 | 10 | 2.3 | 10 | 2.6 |
| Lymphocytes (10^9^/L) | 12 | 1.4 | 12 | 1.4 | 11 | 1.2 | 10 | 1.3 | 10 | 1.5 |
| Monocytes (10^9^/L) | 12 | 0.4 | 12 | 0.4 | 11 | 0.3 | 10 | 0.4 | 10 | 0.4 |
| Platelets (10^9^/L) | 12 | 128 | 12 | 120 | 11 | 95 | 11 | 91 | 10 | 111 |
| CRP (mg/L) | 12 | 3 | 12 | 9 | 11 | 4 | 11 | 4 | 10 | 3 |
| Albumin (g/L) | 12 | 36 | 12 | 34 | 11 | 34 | 11 | 33 | 10 | 34 |
| PK (INR) | 12 | 1.3 | 12 | 1.3 | 11 | 1.3 | 11 | 1.3 | 10 | 1.3 |
| Bilirubin (μmol/L) | 12 | 17 | 12 | 27 | 11 | 23 | 11 | 21 | 10 | 21 |
| ALT (μkat/L) | 12 | 1.7 | 12 | 1.6 | 11 | 1.8 | 11 | 1.6 | 10 | 1.4 |
| Creatinine (μmol/L) | 11 | 66 | 12 | 63 | 11 | 61 | 11 | 60 | 10 | 64 |
| AFP (μg/L) | 12 | 22.5* | 11 | 17* | 10 | 15.5* | 11 | 20* | 10 | 23* |

*Median
